# Distribution characteristics of cow’s milk-sIgE components in children with respiratory allergic diseases in southern China

**DOI:** 10.1186/s12887-020-1971-z

**Published:** 2020-02-24

**Authors:** Huimin Huang, Wenting Luo, Nili Wei, Xueqing Liang, Peiyan Zheng, Haisheng Hu, Baoqing Sun

**Affiliations:** Department of Allergy and Clinical Immunology, National Clinical Research Center of Respiratory Disease, State Key Laboratory of Respiratory Disease, Guangzhou Institute of Respiratory Health, The First Affiliated Hospital of Guangzhou Medical University, 151 Yanjiangxi Road, Guangzhou, 510120 Guangdong China

**Keywords:** Respiratory allergic diseases, Cow’s milk, Component, Specific immunoglobulin E

## Abstract

**Background:**

Cow’s milk (CM) is the main food allergen for toddlers and infants. Presently, studies on CM specific immunoglobulin E (sIgE) sensitization and positive distribution of CM components ALA-, CAS-, and BLG-sIgE are lacking in infants with respiratory allergic diseases, especially in southern China. This study therefore aimed to investigate the distribution of CM sensitization and the relation between its components α-lactalbumin (ALA), β-lactoglobulin (BLG) and casein (CAS) sIgE in children with respiratory allergic diseases in southern China.

**Methods:**

A total of 1839 children (≤12 years) with respiratory diseases and detected CM-sIgE levels were included. Serum samples were collected from the Respiratory Diseases Bioresources Center of the National Center for Respiratory Diseases in southern China from August 2012 to July 2017. ALA-, BLG-, and CAS-sIgE were detected and questionnaires were completed in 103 children.

**Results:**

A total of 36.7% children were positive for CM-sIgE. CM-sIgE levels were higher in asthmatic bronchitis (AB) group than in other allergic respiratory disease groups (all *P* < 0.05). Among the 103 CM-sIgE-sensitized children, 64.08% had a history of family allergies. There were 84.47% of the children who tested positive for two or more sIgE components. The average ALA-, BLG-, and CAS-sIgE levels were 1.91 kU/L, 1.81 kU/L, and 0.62 kU/L, respectively. The CM-sIgE level showed a correlation with BLG-sIgE (*r*_*s*_ = 0.833), ALA-sIgE (*r*_*s*_ = 0.816), and CAS-sIgE (*r*_*s*_ = 0.573) levels (all *p <* 0.001).

**Conclusions:**

In southern China, CM-sIgE levels were higher in children with AB than in those with other respiratory allergic diseases. ALA and BLG were the main allergenic components detected in CM-sIgE-sensitized children with respiratory allergic diseases.

## Highlights

- 36.7% of the children positive for CM-sIgE in southern China
- The positive rare of CM-sIgE levels were higher for asthmatic bronchitis 48.7% than for braochial asthma (34.5%), cough variant asthma (34.4%), combined allergic rhinitis and asthma syndrome (30.3%) and allergic rhinitis (29.1%).
- The positive rare of ALA-, BLG-, and CAS-sIgE were 87.4, 86.4 and 69.9% in children respectively, which was the first study in southern China.

## Background

Globally, the prevalence of food allergies in children is estimated to be 4–7% [1]. Cow’s milk (CM) is one of the most common foods allergen for infants and young children. With the increasing pace of modern life, decreasing rate of breast-feeding, and increasing dependence on formula milk powder, the problem of CM allergy (CMA) has become a serious health concern. In Europe, the prevalence of CMA is above 3% [2], while in America, it is 1.8% among children within 1–7 years of age [3]. In southern China, the rate of self-reported CMA among children aged 1–7 years is 1.9% [4].

Atopy is a risk factor for childhood asthma [5, 6], and our recent research based on real world data shows that there are 28.1% childhood asthma patients who are positive for CM allergen [7]. This may be because sensitization to CM allergens increases the risk of early respiratory allergic diseases in children [8, 9]. Component-resolved diagnostics (CRD) holds promise for improving diagnostic accuracy and has recently been introduced into routine clinical practice [10]. Therefore, early detection of CM sIgE antibodies and analysis of the distribution of CM component sIgE in children with CMA and respiratory allergies are necessary.

The leading components of CM allergen are casein (CAS), α-lactalbumin (ALA) andβ-lactoglobulin (BLG) [11]. CAS accounts for 80% of the CM components and is found in four forms:α-casein, β-casein, γ-casein, and κ-casein. Whey protein accounts for 20% of the CM components and is found as ALA, BLG, bovine serum albumin (Bos D 6), immunoglobulin (Bos D 7) and lactoferrin [12].

Currently, studies on CM sIgE sensitization and positive distribution of CM components ALA-, CAS-, and BLG-sIgE are lacking in infants with different kinds of respiratory allergic diseases, especially in southern China, where there is a large number of potential children with respiratory allergic diseases. According to the survey by Professor Li J [13, 14], the prevalence of physician-diagnosed asthma in children was 6.9%, and that of self-reported allergic rhinitis was 23.2% in southern China (Guangzhou). Thus, the purpose of this study was to investigate sensitization to CM-sIgE and its components (ALA-, BLG-, and CAS-sIgE), as well as to update the spectrum of biological indicators in children with respiratory allergic diseases in southern China.

## Methods

### Ethical considerations

The study was approved by the Ethics Committee of the First Affiliated Hospital of Guangzhou Medical University (Ethical code: GYFYY-2016-73). Serum samples were collected from the Respiratory Diseases Bioresources Center of the National Center for Respiratory Diseases in southern China. All patients had signed the formal written informed consent before their biological samples were entered into the biological resource database. The Ethics Committee of the First Affiliated Hospital of Guangzhou Medical University approved that when using the biological samples from the Respiratory Diseases Bioresources Center of the National Center for Respiratory Diseases, it was only necessary to inform the patients or the parents/guardians of the patients orally. In this study, oral consent was obtained from parents/guardians of the children to participate after they had filled the questionnaires, and then detected serum ALA-, BLG-, and CAS-sIgE of the children.

### Study subjects and questionnaire survey

This wasa prospective study. Children’s serum samples were collected from the Bioresource Bank of the National Center for Clinical Research of Respiratory Diseases from August 2012 to July 2017. We selected 1839 children who met the following criteria: (1) aged ≤12 years; (2) had respiratory allergic disease(s) (diagnosed by respiratory specialists or pediatricians) including bronchial asthma (BA), allergic rhinitis (AR), combined allergic rhinitis and asthma syndrome (CARAS), cough variant asthma (CVA), and asthmatic bronchitis (AB); and (3) were suspected of CMA with CM-sIgE detected. Children with parasitic infection, immune deficiency and other immune diseases were eliminated.

Among them, serum ALA-, BLG-, and CAS-sIgE were detected in a total of 103 CM-sensitized children who completed questionnaires by phone in this study. The questionnaire was answered by parents, and questions included age, gender, birth season, clinical diagnosis, family history and other details **(**Table 1**)**. All the questionnaires were screened strictly based on what we needed to study and ensure the quality of the questionnaire (Additional file 1). Before data entry, we checked and re-checked the original data to remove incomplete or vague questionnaire. Data entry adopted two-way entry method. The researcher who administered the telephone questionnaires also entered the questionnaire information, and this was checked by another researcher.

**Table 1:** Baseline characteristics of 103 CM-sIgE-sensitized children in the study

Characteristic	N	Percentage (%)	Positivity rate of ALA-sIgE (%)	*P*	Positivity rate of BLG-sIgE (%)	*P*	Positivity rate of CAS-sIgE (%)	*P*
Sex								
M	74	71.84	87.84	1.000	83.78	0.339	68.92	0.728
F	29	28.16	86.21		93.1		72.41	
Birth season								
Spr and Sum	42	40.78	95.24	0.069	92.86	0.875	69.05	1.000
Aut and Win	61	59.22	81.97		81.97		70.49	
Age group								
0–2 years	30	29.13	80.00	0.396	96.67	0.081	73.33	0.207
2–3 years	37	35.92	89.19		86.49		59.46	
≥3 years	36	34.95	91.67		77.78		77.78	
Clinical diagnosis								
AB	45	43.69	91.11	0.381	93.33	0.087	66.67	0.528
Non-AB	58	56.31	84.48		81.03		72.41	
Family allergy history								
No history	37	35.92	86.49	0.822	94.59	0.197	70.27	0.918
Both parents	20	19.42	95.00		80.00		70.00	
Only father	19	18.45	84.21		73.68		63.16	
Only mother	18	17.48	83.33		88.89		77.78	
Others	9	8.74	88.89		88.89		66.67	
Duration of breastfeeding								
< 6 months	62	60.19	83.87	0.236	82.26	0.154	72.58	0.466
> 6 months	41	39.81	92.68		92.68		65.85	

*Spr* Spring, *Sum* Summer, *Aut* Autumn, *Win* Winter, *AB* asthmatic bronchitis, *Non-AB* non-asthmatic bronchitis

### Detection of sIgE for CM and its components

The ImmunoCAP system (Thermo Fisher Scientific, Sweden) was used to detect CM-, ALA-, BLG-, and CAS-sIgE. The detection range for sIgE was 0.00–100.00 kU/L. SIgE-positivity was categorized into 6 classes: class 1 (≥0.35 to < 0.70 kU/L), class 2 (≥0.70 to < 3.50 kU/L), class 3 (≥3.50 to < 17.50 kU/L), class 4 (≥17.50 to < 50.00 kU/L), class 5 (≥50.00 to < 100.00 kU/L), and class 6 (≥100.00 kU/L). Children with sIgE levels ≥0.35 kU/L (class 1 or above) were defined as positive cases.

### Statistical analyses

All data were processed using SPSS 19.0 (SPSS, Chicago, IL). Non-parametric quantitative data were presented as medians (with interquartile ranges). The Mann-Whitney test was used as a non-parametric test while the Chi-square test was used for comparison of positivity rates between two groups. Spearman analysis was used for evaluating the levels of sIgE antibodies. *P* values < 0.05 were considered statistically significant.

## Results

### CM-sIgE sensitization in children with respiratory allergic diseases

The median age of the children included in the study was 4.0 (2.0–6.0) years. There were 36.7% cases of CM-sIgE positivity, and the average level of CM-sIgE among the CM-sensitized children was 1.00 (0.56–2.00) kU/L. Among the 1256 males, the CM-sIgE positivity rate was 36.9%, and the average level of CM-sIgE among CM-sensitized males was 1.04 (0.58–2.16) kU/L. On the other hand, among 583 females, the CM-sIgE positivity rate was 36.2%, and the average level of CM-sIgE among the CM-sensitized females was 0.90 (0.54–0.64) kU/L. There were no statistical differences in CM-sIgE levels and positivity rates between the two genders (*P* > 0.05).

### Distribution of serum CM-sIgE in children with different respiratory allergic diseases

**Fig. 1:**
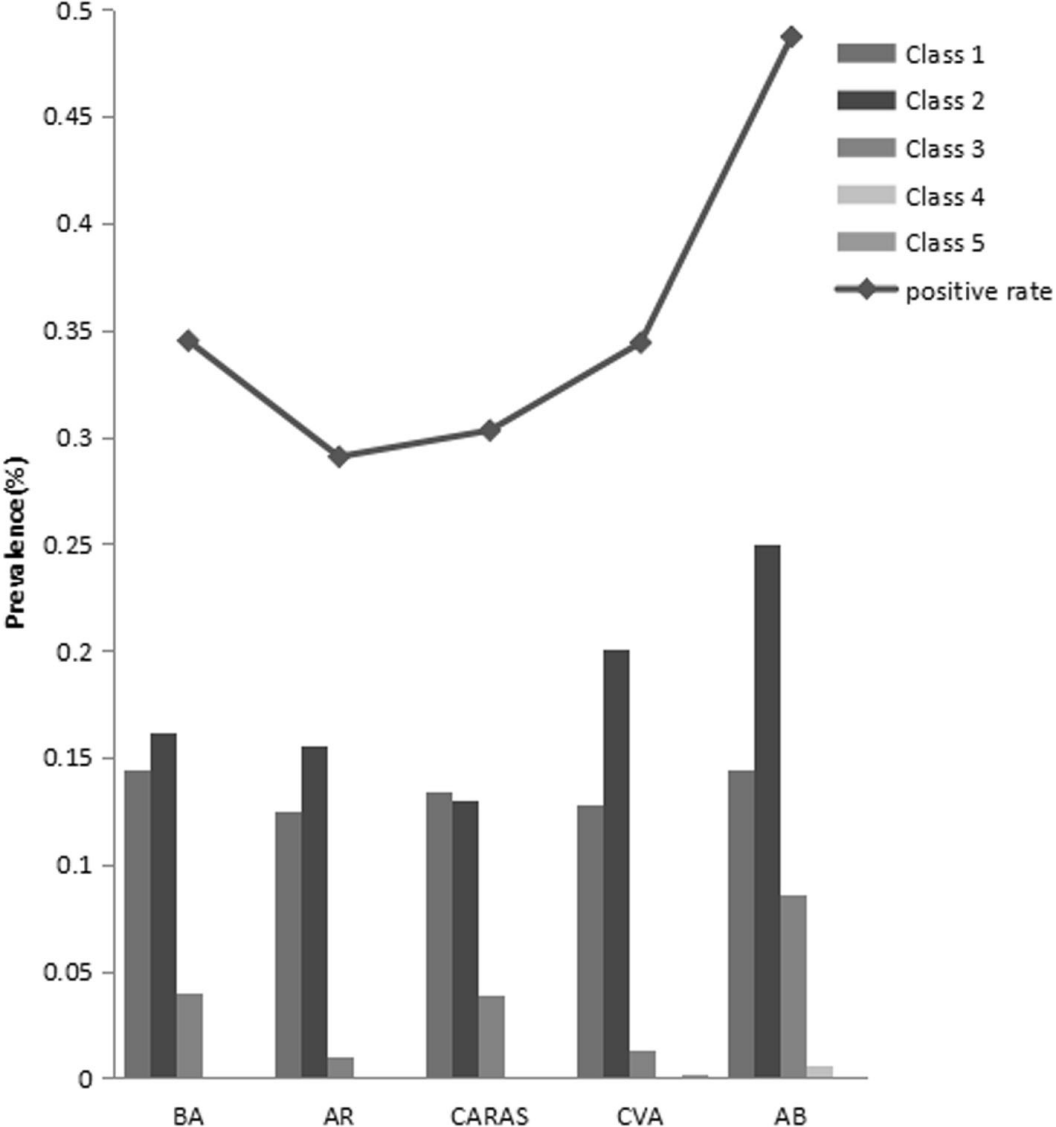
Distribution of CM-sIgE levels in children with different respiratory allergic diseases. For bronchial asthma (BA), there were 403 cases; allergic rhinitis (AR), 296 cases; combined allergic rhinitis and asthma syndrome (CARAS), 208 cases; cough variant asthma (CVA), 468 cases; asthmatic bronchitis (AB), 464 cases

**Fig. 2:**
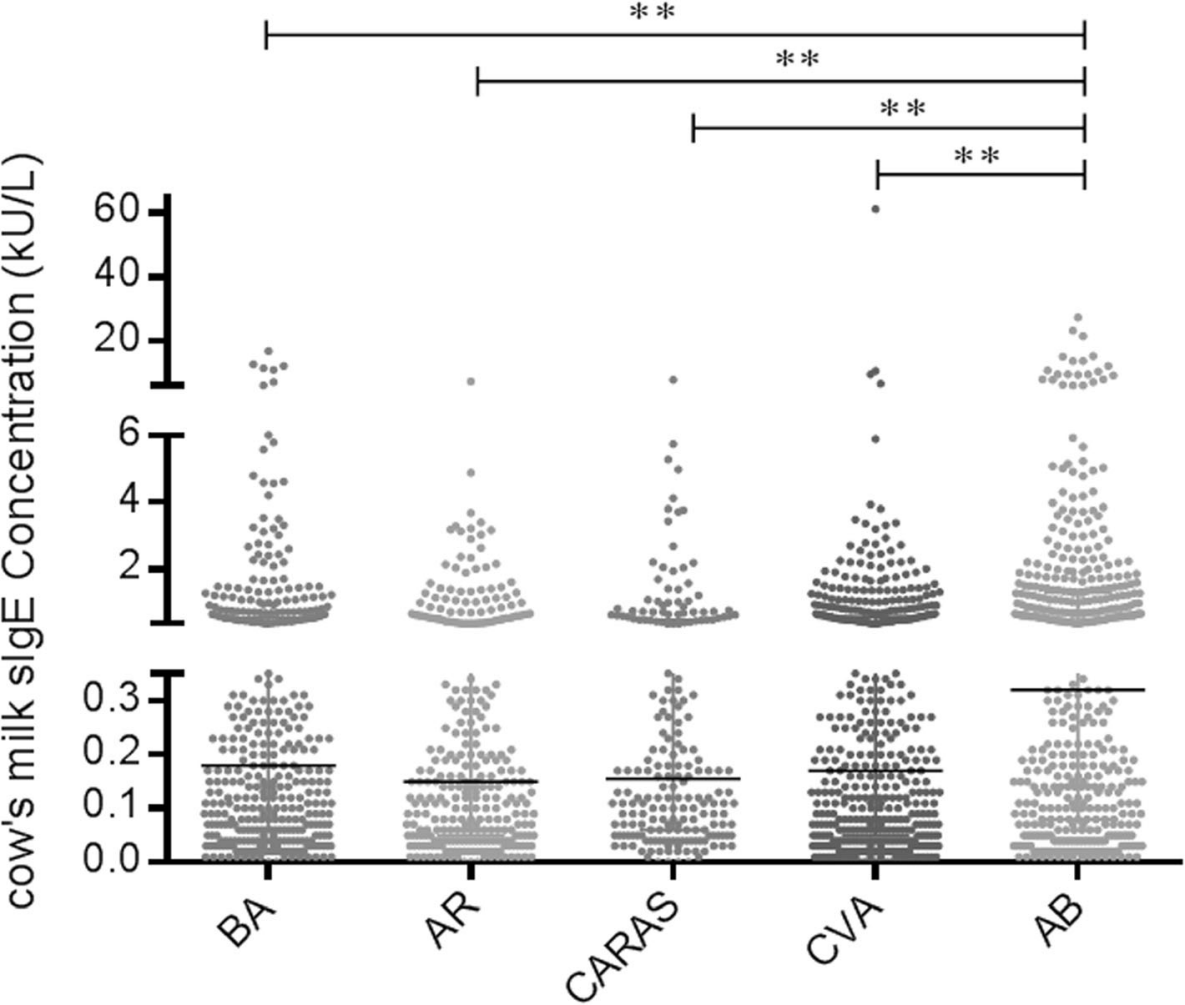
Distribution of the serum level of CM-sIgE in children with different respiratory allergic diseases. Based on the Mann-Whitney test, bronchial asthma (BA), 403 cases; allergic rhinitis (AR), 296 cases; combined allergic rhinitis and asthma syndrome (CARAS), 208 cases; cough variant asthma (CVA), 468 cases; asthmatic bronchitis (AB), 464 cases

The serum CM-sIgE positivity rates in children with different respiratory allergic diseases, from high to low, were: 48.7% in AB group, 34.5% in BA group, 34.4% in CVA group, 30.3% in CARAS group, and 29.1% in AR group. The distribution of CM-sIgE in children with different respiratory allergic diseases was shown in Fig. 1. The proportion of children with class 3 CM-sIgE levels was highest in the AB group (8.6%), followed by the BA group (4.0%), and the AR group (1.0%). Figure 2 shows the distribution of CM-sIgE levels in children with different respiratory allergic diseases. The CM-sIgE level in the AB group was higher than that in the other respiratory allergic disease groups (all *P* < 0.05).

### Relationship between age and serum CM-sIgE levels in children with different respiratory allergic diseases

Negative correlation was found between age and CM-sIgE levels in the 1839 children with respiratory allergic diseases (*r*_*s*_ = − 0.346, *p* < 0.001) in this study. The serum CM-sIgE levels showed negative correlation with age in the BA group (*r*_*s*_ = − 0.325, *P* < 0.001), AR group (*r*_*s*_ = − 0.379, *P* < 0.001), CARAS group (*r*_*s*_ = − 0.446, *P* < 0.001), and CVA group (*r*_*s*_ = − 0.385, *P* < 0.001), but not in the AB group.

### Distribution characteristics of serum CM components in children with respiratory allergic diseases

The questionnaire was completed for 103 children in whom CM components were detected. The median age of the children was 2.0 (1.4–3.0) years, and the average CM-sIgE level was 3.40 (1.46–5.75) kU/L. The basic clinical data for all the children were summarized in Table 1.

A family history of allergies was noted in 64.08% of the children. The ALA-sIgE positivity rate was higher in children who were born in Spring and Summer compared to those born in Autumn and Winter (*P* = 0.069). The BLG-sIgE positivity rate in the AB group was higher than that in the non-AB group (*P* = 0.087). The positivity rate of BLG-sIgE showed a negative correlation with age (*P* = 0.081). However, the above differences were not reach statistically significant.

**Fig. 3:**
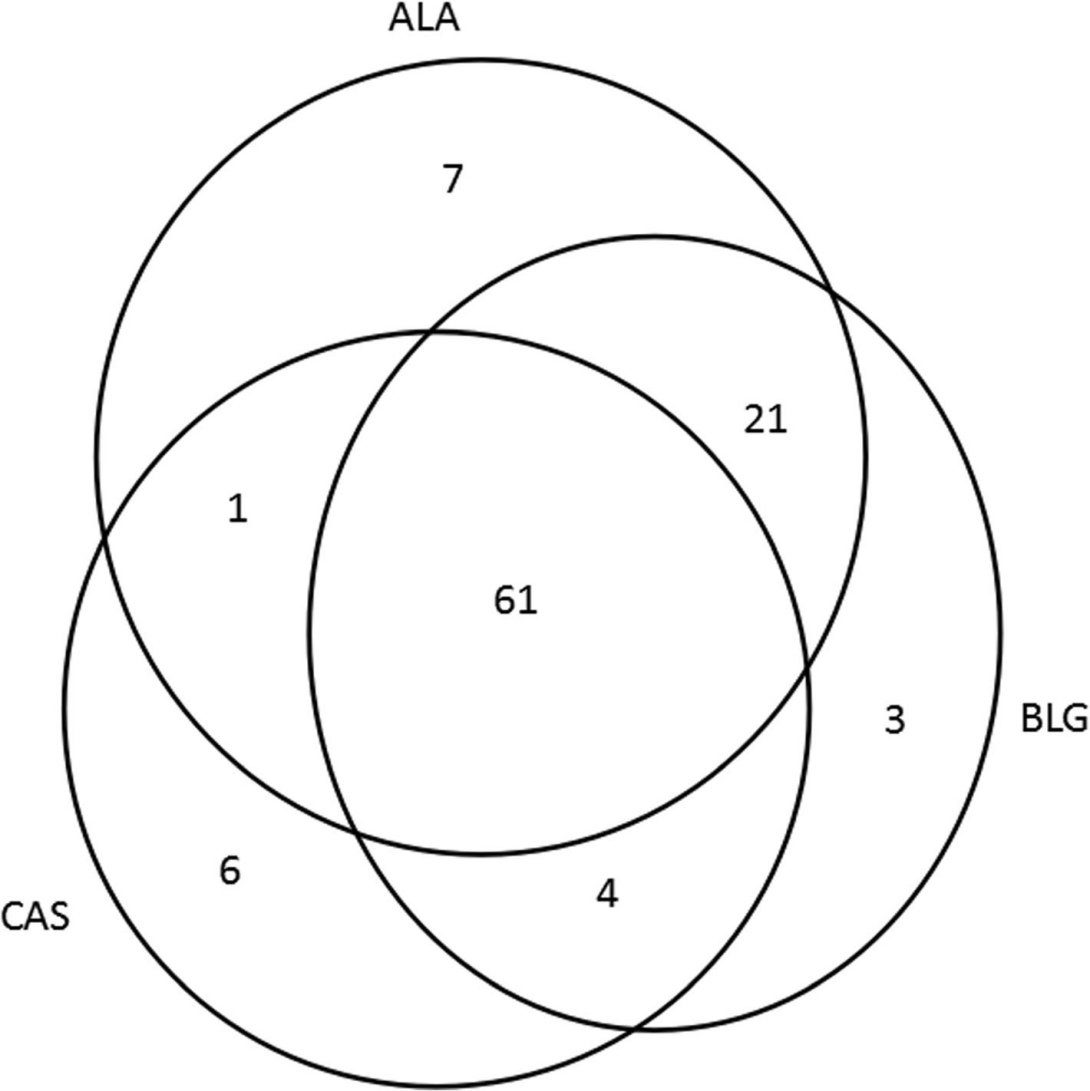
Distribution of cases of sensitization to each CM component. ALA: α-lactalbumin; BLG: β-lactoglobulin; CAS: casein

**Fig. 4:**
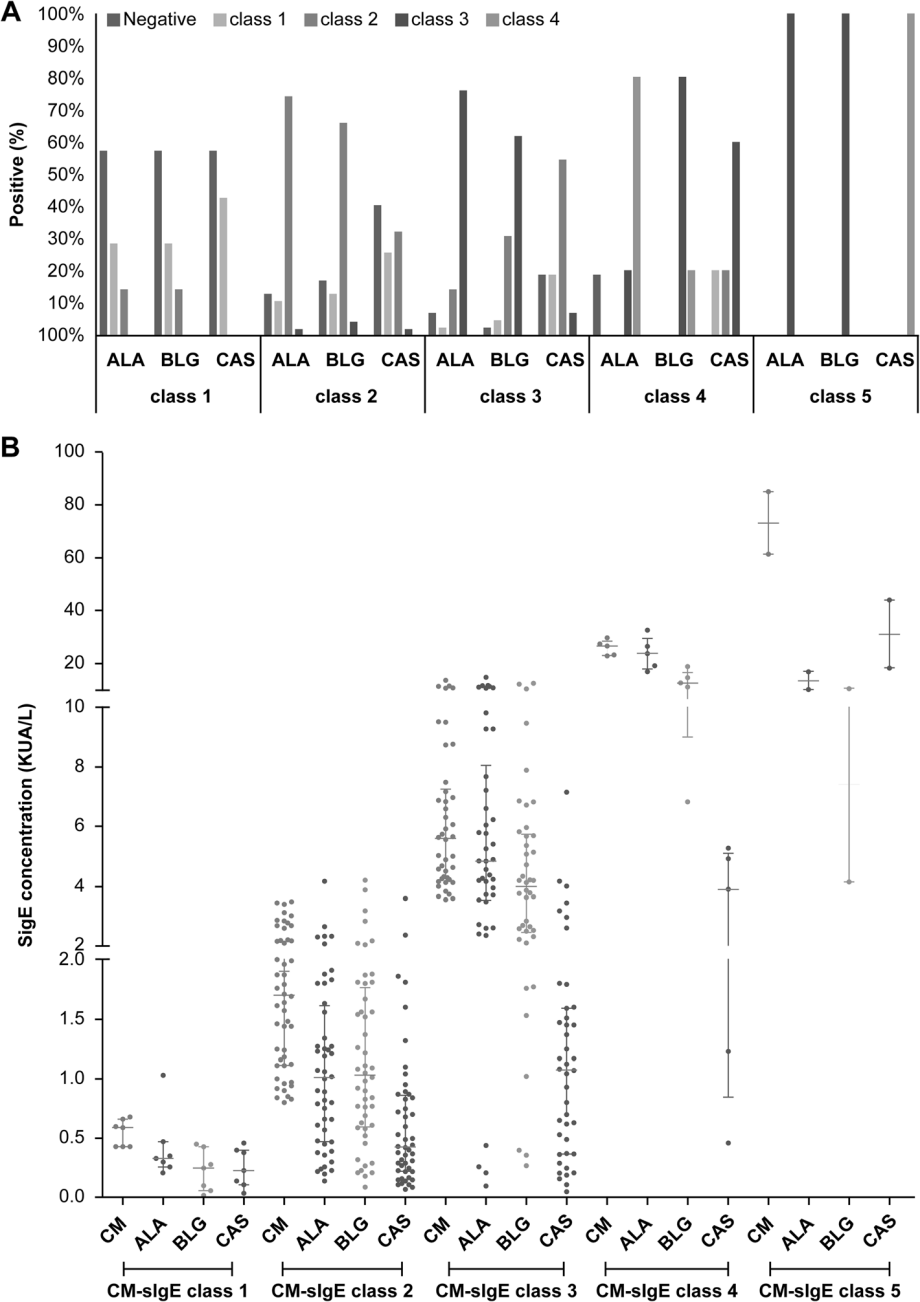
Class and level distribution of the three components of sIgE at different CM-sIgE levels. **a** Class distribution of the three components of sIgE in the different CM-sIgE classes, and **b** Level distribution of ALA-, BLG-, and CAS-sIgE in the different CM-sIgE classes

Among the 103 children, the positivity rate was 87.38% for ALA-sIgE, 86.41% for BLG-sIgE, and 69.90% for CAS-sIgE. Positivity for not less than two and for three sIgE components was seen in 84.47 and 59.22% of the cases, respectively (Fig. 3). Based on the detected CM-sIgE levels, most of the cases belonged to class 2 and 3 (class 1, 6.80%; class 2, 45.63%; class 3, 40.78%; class 4, 4.85%; and class 5,1.94%). ALA-sIgE and BLG-sIgE levels accounted for the highest proportions in classes 2 and 3 (class 2, 40.78 and 42.72%, respectively; and class 3, 34.95 and 33.01% respectively), followed by CAS-sIgE levels in class 1 (23.30%) and class 2 (37.86%). While CM-sIgE levels reached class 5, ALA- and BLG-sIgE levels were class 3, and CAS-sIgE levels were class 4 (Fig. 4a).

The average ALA-, BLG-, and CAS-sIgE levels in CM-sIgE-sensitized children were 1.91 (0.66–5.24) kU/L, 1.81 (0.77–4.19) kU/L, and 0.62 (0.27–1.32) kU/L, respectively. CM-sIgE levels were higher than ALA-sIgE (*z* = − 2.439, *P* = 0.015), BLG-sIgE (*z* = − 3.228, *P* = 0.001), and CAS-sIgE (*z* = − 8.726, *P* < 0.001) levels. ALA-sIgE and BLG-sIgE levels were higher than CAS-sIgE levels (*z* = − 5.720, *P* < 0.001 and *z* = − 5.582, *P* < 0.001, respectively) (Fig. 4b).

### Correlation between levels of CM-, ALA-, BLG-, and CAS-sIgE

**Fig. 5:**
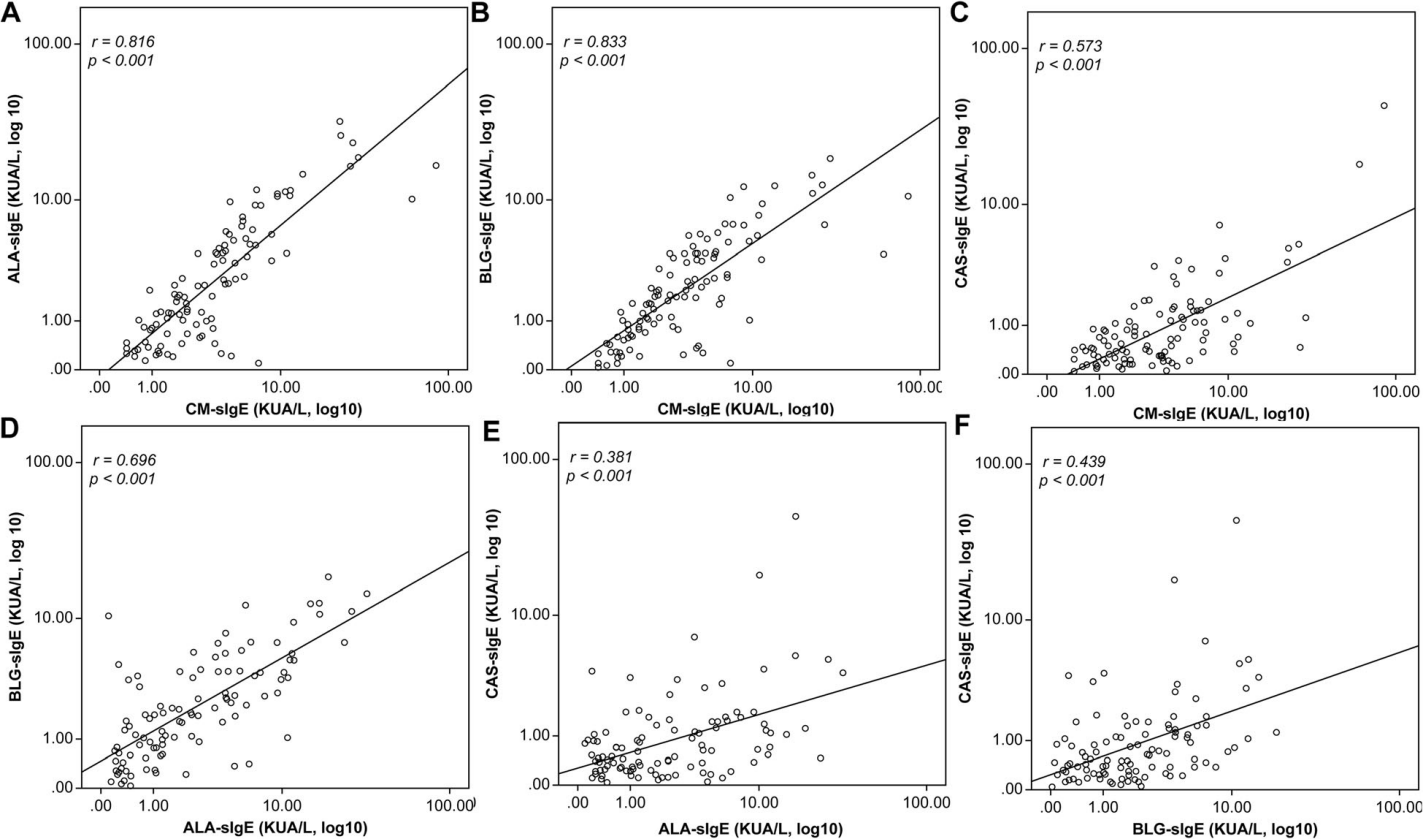
Correlation between levels of CM-, ALA-, BLG-, and CAS-sIgE. This figure shows the correlation between the levels of CM-sIgE and (**a**) ALA-sIgE (*r* = 0.816, *p* < 0.001), (**b**) BLG-sIgE (*r* = 0.833, *p* < 0.001), and (**c**) CAS-sIgE (*r* = 0.573, *p* < 0.001). Also shown was the correlation between the levels of (**d**) ALA-sIgE and BLG-sIgE (*r* = 0.696, *p* < 0.001), (**e**) ALA-sIgE and CAS-sIgE (*r* = 0.381, *p* < 0.001), and (**f**) BLG-sIgE and CAS-sIgE (*r* = 0.439, *p* < 0.001)

With an increase in CM-sIgE levels, the levels of each CM components showed an increasing trend (Fig. 5). Based on the Spearman correlation analysis, the correlation between CM-sIgE and BLG-sIgE was the highest, while that between CM-sIgE and CAS-sIgE was the lowest. Among the three CM components, the correlation between ALA-sIgE and BLG-sIgE was the highest, followed by that between BLG-sIgE and CAS-sIgE, while it was lowest between ALA-sIgE and CAS-sIgE.

## Discussion

Children with infantile food allergy (FA) tend to develop asthma and allergic rhinitis later in life, and this is referred to as the “allergic march” [15]. Studies have shown that children with FA are at an increased risk of developing asthma and decreased lung functions [16–18]. CM is a major cause of food allergies in infants and toddlers and may be related to the occurrence and development of respiratory allergic diseases. This study investigated the positive distribution of CM-sIgE and its components in children with respiratory allergic diseases and found a positivity rate of 36.7%. The positivity rates for ALA-, BLG-, and CAS-sIgE in the CM-sIgE-sensitized children were 87.38, 86.41, and 69.90%, respectively. Tomac et al. [19] reported that early allergen sensitization in infants, especially CMA, was the most important risk factor for subsequent wheezing attacks. In this study, CM-sIgE levels in children with AB were higher than those in children with other respiratory allergic diseases. Lopez et al. [20] have reported that the CM-sIgE level in 12-month-old infants with recurrent wheezing was higher than that in non-wheezing infants. Therefore, clinicians need to pay attention to CMA in children with AB.

In this study, most of the CM-sensitized children reacted to two or more CM components at the same time, which was consistent with the results of D’Urbano et al. [21]. In Taiwan, ALA was shown to be the major sensitizing component in CM-sensitized children with allergic diseases [22]. However, in this study, from southern China, the major sensitizing components were found to be ALA and BLG, with ALA-sIgE and BLG-sIgE levels being higher than CAS-sIgE levels. This difference in the prevalence of CM components may be due to the different types of diseases in the patients [23], thereby indicating different patterns of CM protein sensitization in different diagnoses of CMA. In different regions, dietary structure of children’s milk sensitization model is different. According to Li J et al. [24], the positivity rate of CAS-sIgE was higher than that of ALA-sIgE and BLG-sIgE in children with CMA in northern China.

The positivity rate of CM-sIgE has also been found to decrease with age. Rona et al. [25] have reported that the incidence of self-reported milk allergies is 6–7% in children and 1–2% in adults. More than 50% of children with CMA will become CM tolerant by 5 years of age [26]. With the increase in age, the positivity rate for BLG-sIgE declined, though there were no significant differences among the different age groups probably due to the sample size, suggesting that BLG-sIgE may be the main component of CM-sIgE that decreases with age.

We found that the level of three components CM-sIgE increased with an increase in levels of CM-sIgE. ALA- and BLG-sIgE levels were highly correlated with CM-sIgE levels. Though the CM components of sIgE cannot be used as diagnostic indices of CMA, higher ALA-, BLG-, and CAS-sIgE levels are associated with lower success rate of oral immunotherapy [27], which suggests that the distribution of sIgE could predict the outcomes of CMA.

Although most children could tolerate CM as they grew older, some others had CMA that persisted for their whole lives. The positivity rates and levels of CAS-sIgE were lower than those of ALA-sIgE and BLG-sIgE. However, the recognition of casein fragments by IgE is related to persistent CMA [28–30], and casein is more heat-resistant than whey protein. Allergy to baked milk powder has also been found to be a marker of severe and persistent CMA [31]. Therefore, the level of CAS-sIgE could be a predictor of the type and severity of CMA.

Matsui T et al. [32] showed that food sensitization was related to season of birth, and a study showed that infants born in Autumn and Winter were more likely to develop food allergies [33]. In this study, ALA-sIgE positivity rate was higher in children who were born in Spring and Summer compared to those born in Autumn and Winter, but the difference was not statistically significant. It may be related to the differences of population and research indicators.

According to our survey, there was no statistical difference in the positivity rates of ALA-, BLG-, and CAS-sIgE of CM-sIgE-sensitized children between those who were breastfed for more than 6 months and those who were breastfed for less than 6 months. Although some studies had shown that breastfeeding could reduce the risk of milk allergy in infants [34, 35]. Rajani PS et al. [36] showed that the food antigens in human milk can elicit clinical reactions in some, already-sensitized infants. Therefore, mother’s eating habits will affect the development of food sensitization of breast fed infants.

CM sensitization should not be ignored in children with respiratory allergic diseases, especially in those with AB. Clinicians should pay special attention to children with AB and reconsider treatment strategies based on the patient’s symptoms and the history of food intake.

The main limitation of this study was it only detected three CM components sIgE, but failed to analysis the antibody levels of other CM components, the other CM components maybe also a factors causing respiratory diseases in children, which we can’t ignore. The children in this study were from the respiratory diseases Bioresources center of the National Center for Respiratory Diseases in southern China. All the selected children with respiratory allergic diseases, so we did not included the control group. And the sample size of this study needs to be increased. In addition, we will add longitudinal cohort studies in the future, and to assess the “allergic march” in food allergic children with respiratory diseases.

## Conclusion

This is the first study that explored the positive distribution characteristics of CM-sIgE and its components, including ALA-, BLG-, and CAS-sIgE in children with respiratory allergic diseases in southern China. CM-sIgE levels in children with AB were higher than those in children with other respiratory allergic diseases. ALA and BLG were the main allergenic components detected in CM-sIgE-sensitized children with respiratory allergic diseases.

## Supplementary information

**Additional file 1.** Questionnaire.

## Data Availability

The data that support these findings are available on reasonable request from the corresponding author Baoqing Sun. Data are not publicly available due to concerns regarding research participant privacy.

## Abbreviations

AB: Asthmatic bronchitis
ALA: α-lactalbumin
AR: Allergic rhinitis
Aut: Autumn
BA: Bronchial asthma
BLG: β-lactoglobulin
CARAS: Combined allergic rhinitis and asthma syndrome
CAS: Casein
CM: Cow’s milk
CMA: Cow’s milk allergy
CVA: Cough variant asthma
FA: Food allergy
sIgE: Specific immunoglobulin E
Spr: Spring
Sum: Summer
Win: Winter
