# Supplementary material for: Distribution characteristics of cow’s milk-sIgE components in children with respiratory allergic diseases in southern China
Source: BMC Pediatr. 2020 Feb 24;20:88. doi: 10.1186/s12887-020-1971-z (PMC7038540; doi:10.1186/s12887-020-1971-z)
Supplement: Supplementary file 1 — Additional file 1. Questionnaire. [file 12887_2020_1971_MOESM1_ESM.docx]

**Questionnaire**

Questionnaire No.：_———————_  Date of Form-filling: _———————_

Name: _———————_  Sex: _———————_ Age: _———————_  Clinical diagnosis: _———————_

1. Which season was your child born in？

口Spring 口summer 口autumn 口winter

2. Is anyone in your family suffering from allergic diseases？(If select yes, please continue to complete the next question)

口Yes 口No

3. Does the child's family member have the following allergic diseases?

|  | Food allergy | Allergic dermatitis | Allergic rhinitis | Bronchial asthma | Ocular allergy | Allergic enteritis | Others |
| --- | --- | --- | --- | --- | --- | --- | --- |
| Father |  |  |  |  |  |  |  |
| Mother |  |  |  |  |  |  |  |
| Grandparents |  |  |  |  |  |  |  |
| Brothers and sisters |  |  |  |  |  |  |  |

4. Did your child breastfeed after birth?

口Yes 口 No

5. If so, how long has your child been breastfed?

口< 6 months 口>6months

Thank you for taking time to fill out this questionnaire！
